# Segregation of age-related skin microbiome characteristics by functionality

**DOI:** 10.1038/s41598-019-53266-3

**Published:** 2019-11-14

**Authors:** Hye-Jin Kim, Jin Ju Kim, Nu Ri Myeong, Taeyune Kim, DooA Kim, Susun An, Hanbyul Kim, Taehun Park, Sue Im Jang, Jae Ho Yeon, Ilyoung Kwack, Woo Jun Sul

**Affiliations:** 1 Department of Systems Biotechnology, Chung-Ang University, Anseong, Korea; 2 Safety Research team, Amorepacific R&D Center, Yongin, Korea; 3 Skin Research team, Amorepacific R&D Center, Yongin, Korea; 4 Amorepacific (Shanghai) R&I Center, Shanghai, China; 5 Present Address: Chung-Ang University, Anseong, Korea

**Keywords:** Ecological genetics, Microbial ecology

## Abstract

Although physiological changes are the most evident indicators of skin aging by alteration of the skin’s structure and function, we question whether skin aging is also affected by the structure and assembly process of the skin microbiome. We analysed the skin microbiomes of 73 healthy Chinese women in two age groups (25–35 years old and 56–63 years old) using 16S rRNA gene amplicon sequencing; the overall microbiome structure was significantly different between the two age groups. An analysis using ecological theory to evaluate the process of microbial community assembly processes revealed that the microbiomes of the older group were formed under a greater influence of the niche-based process, with the network of microbes being more collapsed than that of the younger group. Inferred metagenomic functional pathways associated with replication and repair were relatively more predominant in the younger group whereas, among the various metabolism-related pathways, those associated with biodegradation were more predominant in the older group. Interestingly, we found two segregated sub-typing patterns in the younger group which were also observed in the skin microbiomes of young Chinese women living in four other cities in China. The results of our study highlights candidate microbes and functional pathways that are important for future research into preventing skin aging and which could lead to a comprehensive understanding of age-related skin microbiome characteristics.

## Introduction

Skin aging is a natural and inevitable process caused by structural and functional changes in skin cells due to biological age and extrinsic causes (e.g. exposure to ultraviolet radiation and pollution, and poor nutrition)^1^. Although the baseline skin aging rate is determined by an individual’s biological age, it is difficult to clearly separate the intrinsic and extrinsic causes of skin aging, including age spots, wrinkles, sagging, loosening and dryness. In addition, as the area where the epidermis and dermis come together is flattened, the skin becomes fragile and more easily bruised. These typical changes in skin aging are considered a multi-factorial process that can be accelerated by various environmental, lifestyle and/or socioeconomic causes.

Since 1950, the population growth rate of individuals aged ≥60 years old has exceeded the growth rate of the overall global population. With the increase in average life expectancy, the overall proportion of the elderly has been increasing and appropriate care for their skin has become a priority for skin health. Like other human systems, the skin undergoes many age-related changes despite its incredible durability^2,3^. Most people mainly view skin aging as a noticeable and unwelcome physiological change, but the symptoms arise from more complex changes underlying the aging process. Decreased epidermal thickness; reduced water content, fat emulsion and lipid content; and changes in the amino acid composition also indicate skin aging^4–7^. Despite the loss of function in the skin owing to these aging-related events, efforts to alleviate skin aging have been mostly focused on changing or blocking the visible signs of aging because skin aging has long been considered primarily a change in aesthetic appearance rather than indicative of a real functional health problem. However, although skin aging is not a health threat, it can have a detrimental effect on human psychology. Therefore, it is important to investigate its underlying causes and to find possible remedies and preventative measures.

Recent studies have suggested that aside from the gastrointestinal tract, skin harbours the most microbes in the human body^8,9^. This implies that the composition of the skin microbiota can influence an individual’s skin health and condition. The diversity of skin microbial communities depends on various host factors, such as gender, age, health status and geographical location^10–12^. However, striking changes in the skin microbiome have been observed under various host health conditions, such as skin disease or immunodeficiency^13^. Moreover, metagenomic analysis of human skin has shown that its biogeography and individuality shape the temporal dynamics as well as the structural and functional composition of the skin microbiome^8^. Nevertheless, although the relationships of the skin microbiome with the host’s gender, place of residence and various skin diseases have been well studied, that with age remains insufficiently explored.

We focused on the feedback relationship that the changes in the skin caused by aging induce changes in the skin microbiome and the altered skin microbiome further promotes skin aging. In this study, we investigated the age-related characteristics of the microbial community and functional pathways of the skin microbiomes of healthy Chinese women in two age groups (younger women aged 25–35 years old and older women aged 56–63 years old) to assess whether the microbiome plays a key role in the mechanisms of skin aging. We recruited subjects in their 20s–30 s and 50s–60 s whose ages were considered reasonable for the study purposes. Adolescents with relatively high hormonal changes were not included. The microbial composition and community assembly processes (microbial community structure) were characterised and compared between the two age groups using 16S rRNA gene amplicon sequencing. Our objective was to examine differences in the microbial distribution of the skin microbiomes and their functional pathways between younger and older women living in the same area to determine the relationship between the skin microbiome and age.

## Results

### Differences in cheek microbiomes between the two age groups

The cheek microbiomes of 73 healthy female residents of Xi’an who were free from cutaneous disorders were analysed by 16S rRNA gene amplicon sequencing. From the samples obtained from the older women (age 50–60 years old, hereinafter group 50s–60 s), 36,533 average merged sequences were produced and compared with those from a previously reported group of 48 women (age 25–35 years old, hereinafter group 20s–30 s)^12^. UCLUST clustered the merged sequences into 17,324 distinct operational taxonomic units (OTUs) on the basis of ≥97% sequence identity. According to the taxonomical assignments by Ribosomal Database Project II (RDP; http://rdp.cme.msu.edu) classification, the four predominant phyla were Proteobacteria (36.4%, mean relative abundance), Bacteroidetes (22.5%), Firmicutes (18.5%) and Actinobacteria (18.5%), accounting for >90% of the microbiomes of both groups (Fig. S1). The 10 most abundant genera, comprising >57% of the microbiomes, were *Cutibacterium* (formerly *Propionibacterium*), *Chryseobacterium*, *Enhydrobacter*, *Staphylococcus*, *Sphingomonas*, *Bacteroides*, *Acinetobacter*, *Corynebacterium*, *Streptococcus* and *Neisseria* (Fig. S2), which is similar to the composition of a typical cheek microbiome.

**Figure 1:**
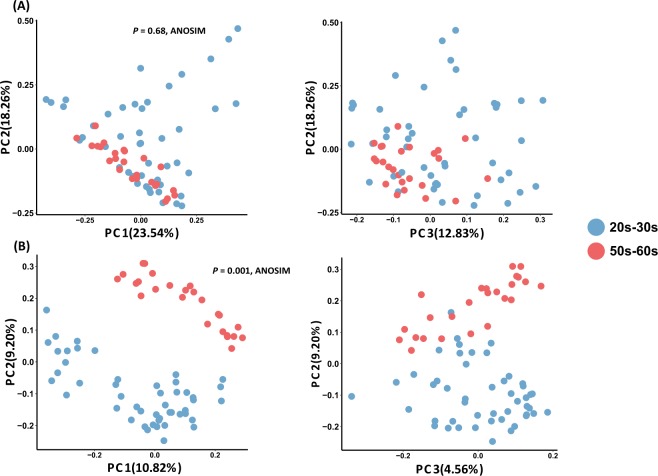
Principal coordinate analysis (PCoA) of skin bacterial communities of group 20s–30 s and group 50s–60 s. PCoA plots of (**A**) Weighted and (**B**) unweighted UniFrac distance based on the 97% operational taxonomic unit (OTU) level of the skin bacterial community compositions. There was a significant difference in composition by age (*P* = 0.001; ANOSIM) explained by PC1 and PC2 with a variance of 10.8% and 9.2%, respectively.

**Figure 2:**
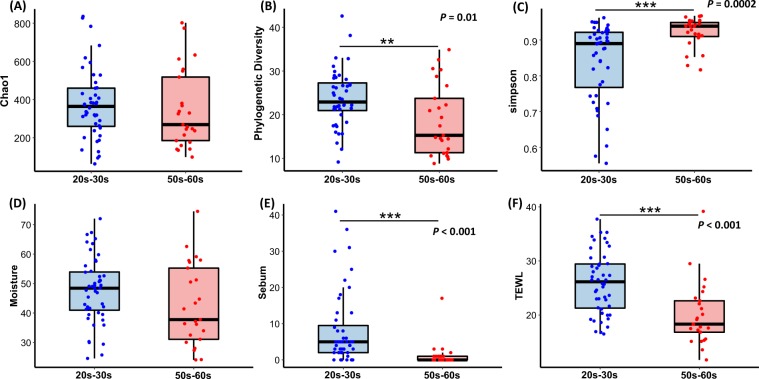
Skin bacterial alpha diversity and skin parameter comparisons between groups 20s–30 s and 50s–60 s. (**A**) Species richness (Chao1) (not significantly) and (**B**) phylogenetic diversity (PD) were higher in group 20s–30 s (*P* < 0.01, Wilcoxon) and (**C**) simpson was higher in group 50s–60 s (*P* < 0.001; Wilcoxon). (**D**) Skin moisture (hydration), (**E**) oil (sebum) and (**F**) transepidermal water loss (TEWL) in both groups. Oil and TEWL were significantly higher in group 20s–30 s (*P* < 0.001; Wilcoxon). The black line and whiskers in the box plot represent the median and range of the lower quartile (25^th^ percentile) and upper quartile (75^th^ percentile) (excluding outliers).

We compared the skin microbiome structure of the two groups using principal coordinates analysis (PCoA) and analysis of similarities (ANOSIM) with both unweighted and weighted UniFrac distances (Fig. 1A). There was a clear separation of the two age groups (*R*^2^ = 0.61, *P* = 0.001; ANOSIM) with presence/absence based on the unweighted UniFrac distance whereas there was no statistical difference based on the weighted UniFrac distance (Fig. 1B). Group 20s–30 s exhibited a higher alpha diversity in both species richness (Chao1) and phylogenetic diversity (PD) (Fig. 2A,B). Additionally, the species evenness was lower in group 20s–30 s (Fig. 2C), indicating that the skin microbiomes of younger women contained dominant members of bacteria. Additionally, the beta diversity, which indicates the heterogeneity within groups, was measured using the unweighted UniFrac distance to compare the range of bacterial PD.

Among the skin physical parameters measured (viz. pH, sebum content, moisture content and transepidermal water loss (TEWL)), the average values of sebum (8.23 and 1.28 in groups 20s–30 s and 50s–60 s, respectively) and TEWL (25.93 and 20.10, respectively) were significantly different (*P < *0.001; Wilcoxon rank-sum test) between the age groups (Fig. 2D–F). In contrast, there was no significant difference in the pH (6.04 and 6.03 in group 20s–30 s and 50s–60 s, respectively) and moisture content (47.72 and 42.49, respectively) between the two groups. Thus, TEWL and sebum are related to skin aging and skin microbiomes.

### Age-dependent microbial signatures of cheek microbiomes

**Figure 3:**
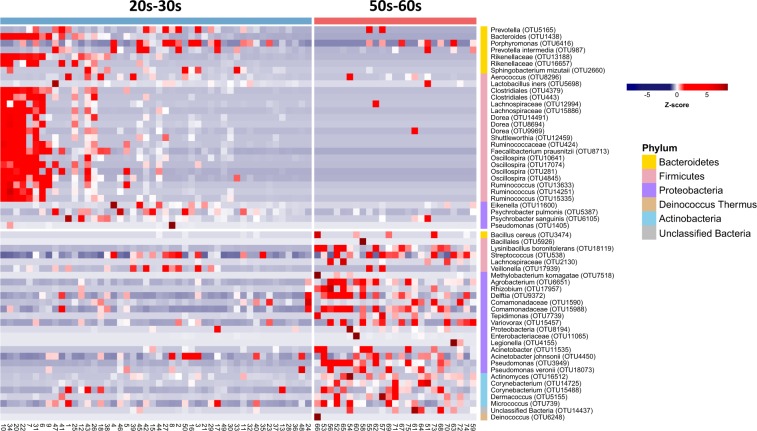
Taxa identification with the most different abundance between groups 20s–30 s and 50s–60 s. The blue colour on the heat map indicates lower abundance and the red colour higher abundance. Bacterial operational taxonomic units (OTUs) were significantly enriched in both groups. The relative abundance of bacterial OTUs represented as groups were detected by LEfSe analysis (*P* < 0.05, LDA > 2.5). The relative abundance was normalized to a Z-score (the number of standard deviations) to represent relative changes across the samples.

We applied the LEfSe (linear discriminant analysis effect size) method to identify the taxonomical biomarker contributing to the age-related variation in skin microbiomes with high stringency (linear discriminant analysis (LDA) >2.5). A total of 58 OTUs were identified as being distinct in either of the two groups; i.e. 30 OTUs in group 20s–30 s and 28 OTUs in group 50s–60 s (Fig. 3 and Table S2). The LEfSe analysis revealed that the Bacteroidetes and Firmicutes phyla were significantly more abundant in group 20s–30 s. Of the OTUs affiliated to Bacteroidetes, those specific to *Bacteroides*, *Alistipes*, *Prevotella*, *Porphyromonas* and *Sphingobacterium* were found in this age group only. Likewise, of the OTUs belonging to Firmicutes, those of *Lactobacillus*, *Aerococcus*, *Oscillospira* and *Ruminococcus* were found only in this group. In contrast, the Proteobacteria and Actinobacteria phyla were more abundant in group 50–60 s. Of the OTUs affiliated to Actinobacteria, those specific to *Micrococcus*, *Corynebacterium*, *Dermacoccus* and *Actinomyces* were found only in the older group, and of the OTUs belonging to Firmicutes, those of *Streptococcus*, *Lysinibacillus* and *Bacillus* predominated.

### Functional profiles of cheek microbiomes predicted by PICRUSt2

**Figure 4:**
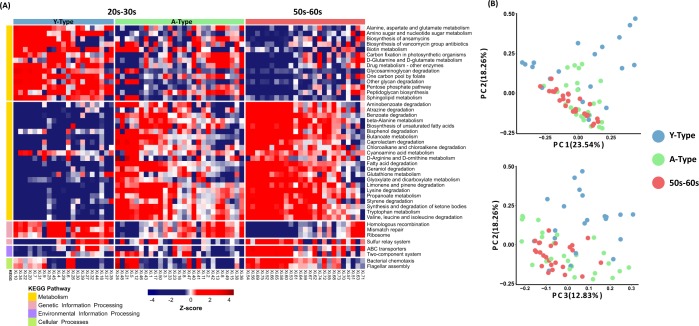
Heatmap of significantly different functional profiles inferred by PICRUSt2 and principal coordinate analysis (PCoA) according to the type of skin microbiome. (**A**) Sub-typing of group 20s–30 s based on PICRUSt2-acquired functional profiles conducted to generate a list of gene categories inferred to be present in the samples. Red colours represent higher abundance and blue colours lower abundance. To represent relative changes across the samples, the relative abundance was normalized to a Z-score. The left-side colour bars indicate the KEGG pathway categories. All pathways within the functional profiles for Metabolism, Genetic Information Processing, Environmental Information Processing and Cellular Processes were significantly different between the two groups (*P *< 0.05; LDA > 2.5). Y-Type is a group 20s–30 s sub-group with a unique pattern when clustered with all 44 significantly different PICRUSt2-acquired pathways and the A-type sub-group had a pattern similar to that of group 50s–60 s. (**B**) PCoA results obtained by clustering group 20s–30 s into two clusters using weighted UniFrac distances.

The functional metagenomic contents inferred using PICRUSt2 analysis were examined to better understand how the bacterial functional profiles differed between the two age groups. On the basis of the LEfSe analysis based on the PICRUSt2 results, we defined 44 differentially abundant KEGG (Kyoto Encyclopaedia of Genes and Genomes) pathways between the two groups. We included those involved in microbial gene functions belonging to the metabolism, genetic information processing, environmental information processing and cellular processes categories (α = 0.05, LDA score >2.5). Among the functional pathways belonging to all of the categories, those predominantly found in group 20s-30s and group 50s-60s were identified (Fig. 4A). Functional pathways belonging to the metabolism category were divided into predominant and non-dominant within each age group. Notably, in the genetic information processing category, homologous recombination, mismatch repair and ribosome pathways were more dominant in the 20s-30s group, while the pathways in the environmental information processing and cellular processes categories were predominant in the 50s-60s group. Interestingly, among the metabolism pathways, more pathways related to degradation were observed in the 50s-60s.

### Skin microbiome functional typing based on functional characteristics

We noticed that the patterns of the 44 differentially abundant functional KEGG pathways (chosen by LDA score >2.5) were similar between some women of group 20s–30 s and the women of group 50s–60 s, whereas other women in group 20s–30 s showed opposite patterns to group 50s–60 s. To clarify these sub-typing patterns in the younger group, we conducted PAM (partitioning around the medoid) clustering based on all of the functional profiles from the PICRUSt2 analysis and then divided group 20s–30 s into Y-type (younger-type; unique to group 20s–30 s) and A-type (aged-type; similar to group 50s–60 s) sub-groups. In particular, it was confirmed that sub-group A-type and group 50s–60 s clearly differed from sub-group Y-type in 36 pathways belonging to the metabolism category (Fig. 4A). In the amino acid metabolism category, pathways related to alanine, aspartate and glutamate metabolism were more predominant in sub-group Y-type whereas those related to lysine degradation, tryptophan metabolism and valine/leucine/isoleucine degradation predominated in sub-group A-type. In the carbohydrate metabolism category, the pathways related to amino sugar and nucleotide sugar metabolism and pentose phosphate pathways were predominant in sub-group Y-type whereas those related to butanoate, glyoxylate and dicarboxylate, and propanoate metabolism were enriched in sub-group A-type. Using all of the PICRUSt2 pathways, we performed a random forest analysis (a supervised classification machine-learning algorithm) and calculated the overall out-of-bag (OOB) error rate (a misclassification error rate) and those between the groups and sub-groups (20s-30s and 50s-60s = 13.7%; Y-type and A-type = 2.08%; Y-type, A-type and group 50s–60 s = 19.18%; Y-type and group 50s–60 s = 4.35%,; A-type and group 50s–60 s = 21.15%; and A-type/50s-60s and Y-type = 2.17%) (Fig. 4A–F). Interestingly, Y-type and A-type showed the lowest OOB error rates (2.08%), and A-type/50s-60s and Y-type also showed the lowst OOB error rated (2.74%). These results indicate that the predicted functional pathway patterns of the A-type sub-group and group 50s–60 s were more similar to each other than to the Y-type sub-group.

**Figure 5:**
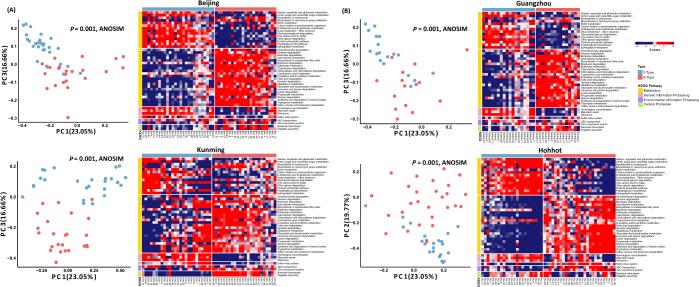
Heatmap of significantly different functional profiles inferred by PICRUSt2 in four cities and principal coordinate analysis (PCoA) according to the type of skin microbiome. PICRUSt2 was performed to confirm that the skin microbiomes of younger women living in Xi’an and in four other cities in China had similar functional profile patterns. On the basis of the 44 significantly different functional pathways between group 20s–30 s and 50s–60 s in Xi’an, PICRUSt2 analysis was performed on the (**A**) Beijing, (**B**) Guangzhou, (**C**) Kunming and (**D**) Hohhot skin samples. Red colours represent higher abundance and blue colours lower abundance. To represent relative changes across the samples, the relative abundance was normalized to a Z-score. The left-side colour bars indicate the KEGG pathway categories. PCoA results obtained by clustering two groups of skin microbiomes in each city are shown on the left-side of the heatmap of each city. PCoA was performed using weighted UniFrac distance matrices.

The bacterial community distribution of the A-type and Y-type sub-groups by functional pathway was also investigated. The skin microbiomes belonging to each type were also separated by PCoA (*P* = 0.001; ANOSIM with weighted and unweighted UniFrac distances) (Fig. 4B). The sub-typing by functional pathways for the Xi’an group 20s–30 s was confirmed against those of the skin microbiomes from young women aged 25–35 years old living in four cities in China (Beijing, Guangzhou, Kunming and Hohhot), whereupon the patterns were found to be similar (Fig. 5).

### Neutral model prediction of the skin microbiomes of the two groups

**Figure 6:**
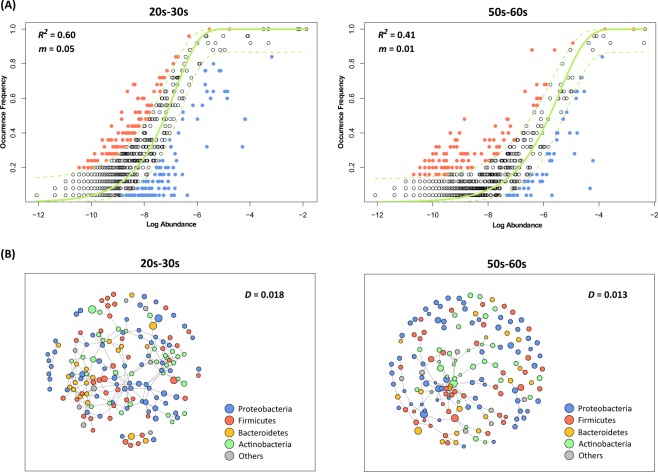
Comparison of the skin microbial community assembly processes and network structures of the two groups. (**A**) The theoretical and observed relationships between the log mean relative abundance of a species and the occurrence frequency were compared to assess the skin microbial community assembly process. Each dot represents a different operational taxonomic unit (OTU) and the solid green line represents the best fit to the neutral model. Dashed lines indicate 95% confidence intervals for the neutral model prediction. OTUs represented by black points within the confidence intervals follow the neutral process. OTUs occurring more frequently than predicted by the model are shown in orange whereas those that occurred less frequently than predicted are shown in blue. The *R*² value indicates the fit for the neutral model. The skin microbial community of group 20s–30 s was a better fit with the neutral model than group 50s–60 s. (**B**) Networks for the two groups were constructed with 168 OTUs with a frequency of >50%. Each node represents OTUs and the node size corresponds to the relative abundance of each OTU. The network density (ratio of the number of edges) of group 50s–60 s (0.012) was lower than that of group 20s–30 s (0.018), suggesting that the skin microbiota network of group 50s–60 s was more collapsed.

To determine the cause of the difference in skin microbiomes between the two age groups, we used the Sloan neutral community model-based dominance test to confirm how the assembly of the skin microbial community proceeded. The neutral model predicts whether a microbial community assembly process follows a neutral or niche-based process by fitting the occurrence frequency and mean relative abundance of the observed bacterial taxa into the neutral model predicted from the metacommunity. The outlying taxa with higher frequencies than those predicted by the neutral model in Fig. 6A are located above the dashed line whereas those taxa with lower frequencies are located below, thereby supporting the hypothesis that the community was assembled through a niche-based process. Consequently, group 20s–30 s (*R*^2^ = 0.60) was a better fit to the neutral model than group 50s–60 s (*R*^2^ = 0.41), indicating that the skin microbial community assembly of the older women was more influenced by a niche-based process. In addition, group 20s–30 s was under a dispersal influence (migration rate (*m*) = 0.05), which may be associated with the neutral process of microbial community assembly. We also confirmed the microbial community assembly process of each group through edge-length abundance distribution (EAD) analysis, using PD to represent the evolutionary history. Both groups had lower EAD values than those of randomly generated null-modelled communities. Moreover, deviations from the prediction (z-scores) were significantly closer to zero for group 20s–30 s (Fig. S3). These results indicate that although the skin microbiomes of both groups were assembled by a niche-based process, the microbial community of group 50s-60s was relatively more influenced by this assembly process.

The skin microbial networks of both groups were constructed via SPIEC-EASI (Sparse InversE Covariance estimation for Ecological Association and Statistical Inference) analysis to examine the overall structure. Co-occurrence network analysis showed that group 20s–30 s had a more complex and stable skin microbial network, with a relatively higher network density (D) than group 50s–60 s (D = 0.018 and 0.013, respectively) (Fig. 6B). These network density tendencies of the two groups can be interpreted as supporting the community assembly process of the taxa leading to each group of microbial networks. The microbial network of group 20s–30 s had a higher edge density and are affected by the neutral process. Conversely, the network of group 50s–60 s appears to have been led by several major taxa formed by niche-based processes (Fig. 6B, where the node size corresponds to the relative abundance of each OTU).

## Discussion

Skin changes are the most evident indicators of aging, manifesting symptoms like dryness due to water loss and increased wrinkle formation. Aside from its association with skin diseases and declined aesthetics in humans^14–16^, it has also been suggested that skin aging is related to changes in the skin microbiome. Shibagaki *et al*.^17^ reported that the skin microbiome was different between a group aged 21–37 years old and one aged 60–76 years old. They also suggested that microbial changes in adult skin were largely influenced by the chronological and physiological skin aging associated with oral bacteria. Although their major microbial compositions were similar to those of our study, their alpha diversity of the cheek microbiomes was higher for the 60–76 years old group, which is in contrast to our results. These differences are explained by the skin microbiome formation process which can be significantly influenced by the urban and living environments, particularly the individual’s residential environment and lifestyle^12^. In addition, several researchers have attempted to investigate the association of skin aging and disease or age-related diseases with microbiomes present at specific sites in the body (e.g. the gut and oral cavity)^18–23^. However, most of these studies examined microbial community differences at the upper taxon levels only (e.g. the phylum level) or suggested the results for specific bacteria or fungi in the context of a particular human disease. A recently reported skin microbiome study in North American subjects has found associations between chronological age, skin aging and specific taxa, and that age is associated with two mutually coexcluding *Corynebacterium* OTUs^24^. However, skin aging variables such as wrinkles and pigmentation spots were correlated with age independently of these taxa. This study also reported that there was no relationship between these taxa and skin aging variables, such as wrinkles and hyperpigmented spots. Although no skin aging variables were measured in our study, further studies that reveal the correlation between groups 20s-30s and 50s-60s (or the A-type and Y-type sub-groups) skin microbiomes and skin aging variables would be very interesting.

In the present study, the skin moisture, sebum content and TEWL were lower in the older women, which was as expected. Aging slows down cellular metabolism over time and the skin will inevitably exhibit aging-related characteristics such as reduced fibroblast activity and collagen synthesis^25–29^. In addition, reduced vascularisation, sweating and sebum secretion cause the skin to dehydrate and become dry. Our results on skin indicators are supported by these reports, suggesting that these physiological changes are correlated with variations in the skin microbiome. As skin microbiome research has progressed, skin parameters such as pH, sebum content, moisture content and TEWL have attracted attention to explain the characteristics of skin microbiomes in various subjects associated with specific skin diseases or aging^24,30^. In our results, sebum content and TEWL were found to be significantly different between the younger and older groups, but PCoA analysis grouped by individual skin parameters (data not shown) showed that the age factor describes the characteristics of the overall skin microbiome better than any particular skin parameter.

The findings of our study clearly show that the skin microbiome structure was significantly different between the two groups, with age being an important influencing factor. Most skin microbiome studies have reported *Cutibacterium* and *Staphylococcus* species as the main bacteria with differential abundance depending on skin diseases such as atopic dermatitis or other skin conditions^31,32^. However, in our study, no significant between-group differences in *Cutibacterium* and *Staphylococcus* abundance were found. In fact, our findings provide a diverse list of microorganisms that show significant differences in abundance between younger and older women.

We identified functional variation on the basis of differences in the microbial communities and through predicted metagenomic pathway analysis. The processes associated with repair and recombination, which were more prevalent in the younger group, should be included in studies related to the various skin characteristics that help skin regeneration or prevent aging. Although the functional differences between the younger and older groups were not experimentally confirmed in our study, the predicted metagenomic pathways with significant differences between the two groups could provide meaningful information on the microbial role in relating skin aging to the skin microbiome. The pattern similarity between the A-type sub-group and group 50s–60 s in the metabolism category was a very interesting finding, as also confirmed by the analysis of other young Chinese women in other major cities in China. On the basis of the clustering together of group 50s–60 s and the A-type sub-group (with the lowest OOB error rate) and their distinctiveness from the Y-type group, we suggest that skin age can be determined by an individual’s skin microbiome rather than chronological age and that younger skin can be achieved by a healthy and well-balanced skin microbiome. Although endogenous aging is the result of genetic factors and changes in the body that occur as part of the normal aging process, it is necessary to understand how lifestyle and environmental factors affect the process as well. From our results, we suggest that understanding the skin microbial community assembly process and the microbial network is more important than controlling the interaction of several major bacteria for exploring the key factors for maintaining younger skin. We suggest that the more niche-based process of microbial community assembly in older women is due to lifestyle and activity differences between the two groups. In the past, people largely maintained cleanliness for keeping the skin healthy, but as skin microbiome research progresses, there is increasing interest in finding ways to help the recovery and regeneration of the skin from the numerous microorganisms living on it. Studies have shown that gender, age, lifestyle, living area, genetic predisposition, diet and drug use (medication intake) are intricately related to the skin microbiome. However, mutual correlation between the skin microbial community and skin aging has not yet been sufficiently studied. Our data herein could be useful for future studies characterising the structure, function and dynamics of the skin microbiome in the aging process. Such knowledge could lead to the design of therapeutic agents for targeting the microbes and their metabolites that contribute to skin aging as well as providing a microbiological interpretation of this process.

## Materials and Methods

### Study design and sample collection

For comparison of age-related skin microbiome characteristics, we recruited 25 women aged 56–63 years old (Table S1) and 48 women aged 25–35 years old who had been involved in a previous study^12^. All of the selected subjects had been living in Xi’an (Shaanxi, China) for at least five years. Medical and drug history for each individual was also investigated and subjects with a history of skin disease and those exposed to antibiotics in the last six months were excluded. To avoid the effects of cosmetics, all subjects refrained from applying cosmetics such as lotions or creams. In addition, we surveyed the amount and frequency of use of facial cosmetics per week by the subjects. The skin condition of each woman, as indicated by the moisture content, sebum level, surface pH and TEWL, was measured. The skin moisture and sebum levels were measured using a Corneometer® CM 825 and Sebumeter® SM 815 (Courage + Khazaka Electronic GmbH, Cologne, Germany), respectively, and expressed in arbitrary units^33^. The skin surface pH was measured using a skin pH-meter® PH905 (Courage + Khazaka Electronic GmbH) and the TEWL was measured using a Vapometer® (Delfin Technologies, Kuopio, Finland). Microbial samples were collected in April 2015 in temperature- and humidity-controlled rooms by swabbing a 2 × 2 cm area of the cheek with sterile Catch-all Sample Collections Swabs (QEC091H, Epicentre, Madison, WI, USA). The swab heads with the collected samples were transferred to screw-capped tubes and stored at −80 °C until genomic DNA (gDNA) extraction. The study protocol was approved by the internal review board of Xijing Hospital (KY20150527-3) and all methods were carried out in accordance with relevant guidelines and regulations. Written informed consent was obtained from all participants in this study.

### Bacterial DNA extraction

Bacterial gDNA extraction was carried out using the gram-positive bacterial cell lysate procedure of the PureLink® Genomic DNA Mini Kit (Life Technologies, Carlsbad, CA, USA). In brief, a lysis buffer containing 20 mg/mL lysozyme was added to each swab sample, after which the tube was vortexed briefly to obtain the lysate. Proteinase K was added at a volume equivalent to one-tenth of the lysis buffer, followed by 445 µL of genomic lysis/binding buffer. Next, two stainless steel beads (5 mm, Qiagen, Hilden, Germany) were placed in each tube and then bead-beating was performed for 1 min using a Bead Beater 16 device (Bio Spec Products Inc., Bartlesville, OK, USA). The tubes were then cooled on ice for 10 min and incubated at 55 °C for 30 min. Finally, after a washing process, the gDNA was extracted by elution with 30 µL of PureLink® Genomic Elution Buffer and stored at −20 °C until sequencing. The concentration and purity of the gDNA were measured using a NanoDrop 2000 spectrophotometer (Thermo Fisher Scientific Inc., Waltham, MA, USA).

### Polymerase chain reaction (PCR) and 16S rRNA gene sequencping

For each gDNA sample, the v4–v5 region of the 16S rRNA gene was amplified using the 518F-926R primer fused with a barcode (N701-N715/S502-S511). The forward primer included the Illumina sequencing primer (5′-TCG TCG GCA GCG TCA GAT GTG TAT AAG AGA CAG CCA GCA GCY GCG GTA AN-3′) and the reverse primer included the Illumina pre-adapter (5′-GTC TCG TGG GCT CGG AGA TGT GTA TAA GAG ACA GCC GTC AAT TCN TTT RAG T-3′). The amplification reaction comprised initial denaturation at 95 °C for 3 min, followed by 25 cycles of amplification (denaturation at 95 °C for 30 s, annealing at 55 °C for 30 s and elongation at 72 °C for 30 s) and a final extension at 72 °C for 5 min. The amplified products were then purified using AMPure XP beads (Beckman Coulter, High Wycombe, UK). An index PCR was performed under the same conditions as the amplification procedure except that eight cycles of amplification were used. The obtained DNA was subjected to quality assessment using PicoGreen dye and a NanoDrop spectrophotometer. The final purified product was quantified by quantitative PCR (qPCR) according to the qPCR Quantification Protocol Guide of the KAPA Library Quantification kits for the Illumina sequencing platform. Additionally, the product was determined using the LabChip GX HT DNA High Sensitivity Kit (PerkinElmer, Waltham, MA, USA). The final samples were sequenced on the Illumina MiSeq™ platform (Illumina, San Diego, CA, USA) as paired-end (2 × 300 bp) reads.

### Bacterial community analysis

The MiSeq paired-end sequence reads were merged with default parameters (*P*-value of 0.3) using Illumina-Utils (https://github.com/meren/illumina-utils)^34^. The merged sequences were processed using the Quantitative Insights into Microbial Ecology (QIIME) pipeline (v.1.9.1)^35^. A total of 1,666,543 merged sequences were clustered into OTUs at a 97% sequence similarity cut-off using UCLUST (pick_otus.py)^36^. The representative OTU sequences were assigned to a taxonomy at a confidence threshold of 50% using RDP classifier^37^ with reference to the Greengenes database^38^. OTUs classified as chloroplasts and mitochondria were excluded from further analysis. The PyNAST aligner was used for the alignment of representative sequences^39^ and FastTree was used to construct a phylogenetic tree^40^ in QIIME. The samples in the OTU table were rarefied to 2,360 reads per sample and the alpha diversity was estimated with the Chao1 estimator, PD whole tree, observed OTUs and Simpson metrics (multiple_rarefactions.py and alpha_diversity.py). Unweighted and weighted UniFrac distance matrices were calculated from the phylogenetic tree using a QIIME script (beta_diverstity.py). PCoA was conducted to identify the between-group differences in the microbial community composition using UniFrac distance matrices and sample metadata (principal_coordinates.py).

### Identification of genomic features using the LEfSe method

The LEfSe method was used to identify differences in the statistically significant taxonomical and functional features between the two groups^41^. The LEfSe algorithm was used to compare all biomarkers between the two groups using the factorial Kruskal-Wallis rank-sum test. Vectors obtained by comparing the abundance between groups were used as an input to LDA, which produced an effect size. To estimate the effect size of each differently abundant feature, LDA scores of 2.5 and an alpha value of 0.05 for the Kruskal-Wallis test were applied for the OTU analysis and the functional profiling analysis.

### Microbial community assemblage

The assembly of the skin microbial community was assessed by applying the neutral community assembly model described by Sloan^42^. This model is used to estimate the relationship between the frequency of occurrence of taxa in communities and their abundance in the broad metacommunity (the skin microbial communities of all of the samples). Since the dispersal opportunities have increased, the model predicts that the abundant taxa are observed more frequently in the metacommunity whereas the rare taxa observed less often than predicted in the metacommunity are likely to be lost from individual hosts owing to ecological drift. To analyse the neutral community assembly model, all samples were rarefied to 7,000 sequences per sample and OTUs with zero abundance in all samples were excluded from the analysis. To equalise the sample number in both age groups, 25 of the 48 women in the younger group were randomly selected for the analysis. The neutral community assembly model fit is determined by a parameter called the migration rate (*m*), which estimates the influence of dispersal opportunities. The fitting of the model analysis was conducted using a non-linear least-squares fitting program and the R package minpack.lm. The calculation of 95% confidence intervals based on the Wilson score interval around the model predictions was processed using the HMisc package in R^43^.

### Microbial association network analysis

To analyse the skin microbial networks of the two age groups, we selected bacterial OTUs with a frequency of ≥50% and excluded those with an abundance of zeros in the samples of each group; 168 OTUs in each age group were used for the analysis. For each set, we used the SPIEC-EASI framework, a statistical method for the inference of microbial ecological networks in OTU datasets^44^. We ran a network analysis using the sparse neighbourhood algorithm^45^ and model selection using the Stability Approach to Regularization Selection (StARS) method^46^ with a variability minimum lambda (λ) threshold of 0.05%. All steps in the network analysis were processed using the R package SpiecEasi:0.1.2^44^ and the igraph package.

### Prediction of functional profiling by PICRUSt2

The functional profiles from 16S rRNA data were predicted using Phylogenetic Investigation of Communities by Reconstruction of Unobserved States 2 (PICRUSt2) v.2.1.3-b software^47^ which predicts gene family abundance. Using the OTU table from the bacterial community analysis (97% OTU clustering; UCLUST) and representative sequences, predicted functional profiles were obtained using the PICRUSt2 script with default options (picrust2_pipeline.py). Afterwards, we inferred the Kyoto KEGG pathway abundances from the predicted KEGG ORTHOLOGY (KO) abundances with the “–no_regroup” option (pathway_pipeline.py).

### PAM clustering analysis

From the LEfSe analysis of the PICRUSt2 results, 44 functional pathways were found to be significantly different between the two groups of women. Moreover, we found a pattern of sub-groups in the younger age group where different trends for these 44 functional pathways were exhibited. To verify the pattern found in this age group, we performed PAM clustering using the Canberra distance^48^ of previously studied skin samples from young women in four cities in China^12^ with those from our Xi’an young age group. After assigning clusters to each sample using PAM clustering, we visualised the abundance of functional pathways using a heatmap. PCoA of the bacterial community was conducted with weighted UniFrac distance. Random forest analysis was conducted using the group of PAM clustering result. We calculate the out-of-bag error rate (OOB rate) from the entire functional pathways using R package randomForest with 999 trees^49^.

### Statistical methods

Analysis of variance was performed on the pH, moisture, sebum and TEWL data to find statistically significant causes of age-related differences, for which only TEWL showed a significant relationship. ANOSIM was used on the weighted and unweighted UniFrac distance matrices in the QIIME script with 999 permutations (compare_categories.py–method anosim), to estimate the significant difference between the bacterial communities of the two groups. To determine the significance of the alpha diversity, we performed a non-parametric Mann–Whitney U test and Kruskal-Wallis test with wilcox.test and kruskal.test, respectively, in R.

## Supplementary information

Supplementary Figure legends

Supplementary Material and method

Supplementary Tables
