# Supplementary material for: Segregation of age-related skin microbiome characteristics by functionality
Source: Sci Rep. 2019 Nov 14;9:16748. doi: 10.1038/s41598-019-53266-3 (PMC6856112; doi:10.1038/s41598-019-53266-3)
Supplement: Supplementary file 1 — Supplementary Figure legends [file 41598_2019_53266_MOESM1_ESM.docx]

**Segregation of age-related skin microbiome characteristics by functionality**

Hye-Jin Kim^a†^, Jin Ju Kim^a^, Nu Ri Myeong^a^, Taeyune Kim^a^, DooA Kim^a^, Susun An^b^, Hanbyul Kim^b^, Taehun Park^b^, Sue Im Jang^c^, Jae Ho Yeon^d^, Ilyoung Kwack^d^, Woo Jun Sul^a*^

^a^Department of Systems Biotechnology, Chung-Ang University, Anseong, Korea.

^b^Safety Research team, Amorepacific R&D Center, Yongin, Korea.

^c^Skin Research team, Amorepacific R&D Center, Yongin, Korea.

^d^Amorepacific (Shanghai) R&I Center, Shanghai, China.

***Address correspondence to Woo Jun Sul, sulwj@cau.ac.kr**

***Present address: Chung-Ang University, Anseong, Korea**

**Running head:** Age-related characteristics of the skin microbiome

**Supplementary figure legends.**

**Fig. S1. Taxonomical analysis of skin microbiomes at the phylum level.** A total of 34 phyla were found in all the samples. Of these, 10 predominant phyla occupied > 99% in both group 20s–30s and group 50s–60s. The remaining phyla are marked as Others.

**Fig. S2. Taxonomical analysis of skin microbiomes at the genus level.** A total of 983 bacterial genera were found in all of the samples. Of these, 10 predominant genera occupied > 60% in group 20s–30s and 49% in group 50s–60s. The remaining genera are marked as Others.

**Fig. S3. Assessment of the skin microbiome assembly process using edge-length abundance distribution.** Differences in the standard deviation (z-score) between the observed and expected phylogenetic diversities showed that group 50s–60s was more affected by the niche-based process, especially environmental filtering (*P <* 0.05, t-test). The box plot shows the distribution of z-scores in each group. The dashed line indicates the 95% confidence interval and the black line and whiskers in the box plot represent the median and range, respectively, of the minimum and maximum z-scores among the groups.

**Fig. S4. Multidimensional plot of the Random forest analysis**

Multidimensional plot of the proximity matrix calculated from random forest analysis of the functional profiles. We compared **(a)** 20s-30s and 50s-60s, (b) Y-Type and A-Type, (c) Y-type, A-type and group 50s-60s, (d) Y-Type and 50s-60s, (e) A-type and group 50s-60s, and (f) A-Type/50s-60s and Y-Type.
