# Supplementary material for: Segregation of age-related skin microbiome characteristics by functionality
Source: Sci Rep. 2019 Nov 14;9:16748. doi: 10.1038/s41598-019-53266-3 (PMC6856112; doi:10.1038/s41598-019-53266-3)
Supplement: Supplementary file 2 — Supplementary Material and method [file 41598_2019_53266_MOESM2_ESM.docx]

**Segregation of age-related skin microbiome characteristics by functionality**

Hye-Jin Kim^a†^, Jin Ju Kim^a^, Nu Ri Myeong^a^, Taeyune Kim^a^, DooA Kim^a^, Susun An^b^, Hanbyul Kim^b^, Taehun Park^b^, Sue Im Jang^c^, Jae Ho Yeon^d^, Ilyoung Kwack^d^, Woo Jun Sul^a*^

^a^Department of Systems Biotechnology, Chung-Ang University, Anseong, Korea.

^b^Safety Research team, Amorepacific R&D Center, Yongin, Korea.

^c^Skin Research team, Amorepacific R&D Center, Yongin, Korea.

^d^Amorepacific (Shanghai) R&I Center, Shanghai, China.

***Address correspondence to Woo Jun Sul, sulwj@cau.ac.kr**

***Present address: Chung-Ang University, Anseong, Korea**

**Running head:** Age-related characteristics of the skin microbiome

**Supplementary Materials and methods**

**Identification of genomic features using linear discriminant analysis effect size (LEfSe)**

LEfSe was performed in order to identify differences in the statistically significant taxonomical and functional features between the two groups. We followed Biobakery’s LEfSe tutorial (https://bitbucket.org/biobakery/biobakery/wiki/lefse) on Linux with two steps. We generated LEfSe format file from the abundance table (format_input.py) using normalization with 1 million, after which the effect size of each differently abundant features between two groups was estimated with LDA scores of 2.5 and an alpha value of 0.05 for the Kruskal-Wallis test (run_lefse.py).

**Microbial community assemblage**

To estimate the relationship between the frequency of occurrence of taxa in communities and their abundance in a broad metacommunity, we used R code “ismej2015142x4.r” provided by ^1^.

1 Burns, A. R. *et al.* Contribution of neutral processes to the assembly of gut microbial communities in the zebrafish over host development. **10**, 655 (2016).
