# Supplementary material for: Segregation of age-related skin microbiome characteristics by functionality
Source: Sci Rep. 2019 Nov 14;9:16748. doi: 10.1038/s41598-019-53266-3 (PMC6856112; doi:10.1038/s41598-019-53266-3)
Supplement: Supplementary file 3 — Supplementary Tables [file 41598_2019_53266_MOESM3_ESM.docx]

**Segregation of age-related skin microbiome characteristics by functionality**

Hye-Jin Kim^a†^, Jin Ju Kim^a^, Nu Ri Myeong^a^, Taeyune Kim^a^, DooA Kim^a^, Susun An^b^, Hanbyul Kim^b^, Taehun Park^b^, Sue Im Jang^c^, Jae Ho Yeon^d^, Ilyoung Kwack^d^, Woo Jun Sul^a*^

^a^Department of Systems Biotechnology, Chung-Ang University, Anseong, Korea.

^b^Safety Research team, Amorepacific R&D Center, Yongin, Korea.

^c^Skin Research team, Amorepacific R&D Center, Yongin, Korea.

^d^Amorepacific (Shanghai) R&I Center, Shanghai, China.

***Address correspondence to Woo Jun Sul, sulwj@cau.ac.kr**

***Present address: Chung-Ang University, Anseong, Korea**

**Running head:** Age-related characteristics of the skin microbiome

Supplementary Table 1. Sample metadata, number of sequences and alpha-diversity across samples based on rarefaction of 2,360 reads per sample.

^a^ arbitrary units

Supplementary Table 2. A list of OTUs clustering with 97% similarity, which is significantly different between two groups.
